# Guanidinoacetic Acid and Methionine Supplementation Improve the Growth Performance of Beef Cattle via Regulating the Antioxidant Levels and Protein and Lipid Metabolisms in Serum and Liver

**DOI:** 10.3390/antiox14050559

**Published:** 2025-05-08

**Authors:** Simeng Yi, Jinze Wang, Boping Ye, Xin Yi, Abudusaimijiang Abudukelimu, Hao Wu, Qingxiang Meng, Zhenming Zhou

**Affiliations:** 1 State Key Laboratory of Animal Nutrition and Feeding, College of Animal Science and Technology, China Agricultural University, Beijing 100193, China; yisimeng97@163.com (S.Y.); sy20233040882@cau.edu.cn (J.W.); sy20233040883@cau.edu.cn (B.Y.); sy20203040706@cau.edu.cn (X.Y.); abudu18548904186@163.com (A.A.); wu2213@cau.edu.cn (H.W.); qxmeng0624@126.com (Q.M.); 2 Frontier Technology Research Institute of China Agricultural University in Shenzhen, China Agricultural University, Shenzhen 518119, China; 3 College of Grassland Science and Technology, China Agricultural University, Beijing 100193, China

**Keywords:** guanidinoacetic acid, methionine, growth performance, antioxidant capacity, protein and lipid metabolisms, beef cattle

## Abstract

Guanidinoacetic acid (GAA) has been used in ruminant feeding, but it is still unclear whether the exogenous addition of methyl donors, such as methionine (Met), can enhance the effects of GAA. This study investigated the effects of dietary GAA alone or combined with Met on beef cattle growth performance and explored the underlying mechanisms via blood analysis, liver metabolomics, and transcriptomics. Forty-five Simmental bulls (453.43 ± 29.05 kg) were assigned to three groups for 140 days: CON (control), GAA (0.1% GAA), and GAM (0.1% GAA + 0.1% Met), where each group consisted of 15 bulls. Compared with the CON group, the average daily gain (ADG) and feed conversion efficiency (FCE) of the two feed additive groups were significantly increased, and the digestibility of neutral detergent fiber (NDF) was improved (*p* < 0.05). Among the three treatment groups, the GAM group showed a higher rumen total volatile fatty acids (TVFAs) content and digestibility of dry matter (DM) and crude protein (CP) in the beef cattle. The serum indices showed that the contents of indicators related to protein metabolism, lipid metabolism, and creatine metabolism showed different increases in the additive groups (*p* < 0.05). It is worth noting that the antioxidant indexes in the serum and liver tissues of beef cattle in the two additive groups were significantly improved (*p* < 0.05). The liver metabolites related to protein metabolism (e.g., L-asparagine, L-glutamic acid) and lipid metabolism (e.g., PC (17:0/0:0)) were elevated in two additive groups, where Met further enhanced the amino acid metabolism in GAM. In the two additive groups, transcriptomic profiling identified significant changes in the expression of genes associated with protein metabolism (including *PIK3CD*, *AKT3*, *EIF4E*, *HDC*, and *SDS*) and lipid metabolism (such as *CD36*, *SCD5*, *ABCA1*, *APOC2*, *GPD2*, and *LPCAT2*) in the hepatic tissues of cattle (*p* < 0.05). Overall, the GAA and Met supplementation enhanced the growth performance by improving the nutrient digestibility, serum protein and creatine metabolisms, antioxidant capacity, and hepatic energy and protein and lipid metabolisms. The inclusion of Met in the diet was shown to enhance the nutrient digestibility and promote more efficient amino acid metabolism within the liver of the beef cattle.

## 1. Introduction

Guanidinoacetic acid (GAA), an amino acid derivative, serves as a vital substrate in the biosynthesis of creatine within animals [1]. Creatine, in turn, plays a crucial role as an energy-providing compound for skeletal muscle during the growth and maturation phases of animals [2]. Maintaining adequate creatine levels is essential to support optimal growth rates in animals [3,4]. However, the growth phase is characterized by a high demand for creatine, which often exceeds the capacity of endogenous synthesis. Consequently, exogenous supplementation becomes necessary to meet the requirements for accelerated growth [5]. GAA has the advantages of low production cost, high bioavailability, and high stability compared with creatine [6]. In recent years, its use as a growth-promoting additive has been demonstrated in several studies to improve the growth performance in livestock and poultry [7,8,9,10,11], and its functions are not limited to growth promotion; for example, Li et al. [12] found that GAA can improve the antioxidant capacity of the rumen of meat sheep, and Yi et al. [11] further confirmed that GAA can enhance the antioxidant level of Angus cattle serum and optimize nitrogen metabolism efficiency. It is worth noting that the premise for GAA to exert its growth-promoting effect in ruminants is that the body has sufficient methyl donors. Recent studies have suggested that methyl donor restriction may restrict the maximum effect of GAA in beef cattle [11], which suggests that it is necessary to systematically analyze the relevant mechanisms of GAA regulating beef cattle growth.

Methionine (Met) assumes the role of an indispensable amino acid, exerting a critical influence on the growth of the animal organism and making a substantial contribution to creatine biosynthesis within the animal system, providing the indispensable methyl moiety for this synthetic endeavor [13]. It has been reported that supplementing with Met can improve the growth performance of ruminants and positively influence their nitrogen metabolism [14,15]. According to Zhang et al. [9], a mixture of 0.08% GAA and 0.06% Met improved both the growth performance and meat quality in goats. Nevertheless, the study lacked a direct comparison with GAA supplementation administered independently. Currently, limited research has been conducted to evaluate the effects of GAA supplementation, both independently and alongside Met, on beef cattle growth performance. Determining whether Met inclusion can further enhance the benefits of GAA in beef cattle is particularly important.

The synthesis of creatine from GAA is a multi-tissue process. In animals, GAA is synthesized in the kidneys and transported via the bloodstream to the liver, where it serves as a precursor for creatine production. Following synthesis, creatine is secreted by the liver into circulation, enabling its delivery to various tissues [1]. Thus, preserving normal liver function is of utmost importance for the efficient synthesis and metabolism of creatine in animals. Notably, it has been estimated that the liver utilizes approximately 20% of the body’s overall energy resources to sustain its diverse array of metabolic processes, encompassing glucose metabolism, protein metabolism, and lipid metabolism [16]. As a key metabolic organ in ruminants, the liver possesses the ability to modulate the metabolic functions of the animal organism, especially protein metabolism, in response to extrinsic stimuli, such as dietary modifications, which are inextricably linked to the animal’s growth dynamics [17]. Therefore, whether the exogenous addition of GAA and Met would cause changes in the liver metabolic functions is also worth exploring in depth. The primary objective of this study was to evaluate how GAA influences beef cattle growth metrics and to determine whether incorporating Met could enhance the outcomes of GAA supplementation. This study was the first to systematically compare the effects of GAA supplementation alone and with GAA + Met combined supplementation on growth performance and liver metabolism in beef cattle, as well as combine transcriptome and non-targeted metabolome analysis to reveal its possible molecular regulatory mechanism. This not only expands the theoretical basis for the application of GAA but also provides a new scientific basis for improving the nutritional efficiency and production performance of beef cattle.

## 2. Materials and Methods

This study obtained ethical approval from China Agricultural University with the number AW81404202-1-4.

### 2.1. Experimental Material

The GAA employed was characterized by white, uncoated granules with an efficacy content of GAA at or above 96%. Meanwhile, the utilized Met comprised yellow, coated particles, featuring palm fat powder as the coating agent, and had an effective Met content of no less than 60%. The degradation rate of guanidine acetate and methionine in the rumen and the release rate in the small intestine are shown in Figure S1.

### 2.2. Experimental Design, Animals, and Diets

Forty-five Simmental bulls, weighing 453.43 ± 29.05 kg, were randomly allocated to three treatment groups according to body weight, with each group containing 15 bulls. In each treatment group, 15 bulls were randomly assigned to three separate pens with 5 bulls in each pen. Each pen was equipped with an automatic feed-weighing system, which can identify the electronic ear tag number of each bull and record the feed intake of each bull every day. This research included three groups: a control group (CON) without any supplements; a GAA-supplemented group (GAA) that received 1 g/kg of GAA in the dry matter (DM) diet; and a combined supplementation group (GAM) that received both GAA and Met at 1 g/kg in the DM diet. The dosage levels were determined from earlier studies [11]. The trial spanned 140 days, with an initial 15-day adaptation period, followed by 125 days of monitoring. The total mixed ration (TMR) formulation for beef cattle growth was formulated according to the NASEM [18] (Table S1). During the experimental period, the cattle received the TMR diet twice daily at 08:00 and 16:00. GAA and Met were fed by premixing them with concentrate feed and then thoroughly mixing the concentrate feed that contained additives with roughage. All cattle received adequate feed and water throughout the experiment.

### 2.3. Sample Collection

During this research, the feed specimens were gathered biweekly, subjected to drying at 65 °C over a 48 h period, and subsequently placed in plastic pouches for nutrient evaluation. During the final three days, daily pre-feeding samples were also collected and processed identically. After the experiment concluded, rectal fecal samples from each bull were collected, placed in dry aluminum containers, and stabilized with 10% tartaric acid before being dried at 65 °C to an air-dry state (*n* = 15). All processed samples—both fecal and feed—were kept at 4 °C in plastic bags for a later analysis of the apparent digestibility.

The initial (IBW) and final body weights (FBW) were measured by weighing the cattle before morning feeding at the experiment’s commencement and conclusion. The average daily gain (ADG) was subsequently derived from these weight data (*n* = 15). An automated feed-weighing system was employed to track each bull’s daily dry matter intake (DMI) (*n* = 15). Furthermore, the feed conversion efficiency (FCE) was determined by computing the ratio of the ADG to the DMI (*n* = 15).

Upon completion of the trial, rumen fluid was extracted from each bull through oral intubation and filtered using four layers of gauze. The filtered fluid was divided into 15 mL and 50 mL centrifuge tubes, with the latter used for pH assessments (*n* = 15). All rumen fluid samples were promptly stored in an ultra-low-temperature freezer at −80 °C. Blood samples were collected from the tail vein using vacuum tubes for further analysis (*n* = 15). After clotting for 30 min at the ambient temperature, the blood was spun at 3000 rpm for 10 min to obtain the serum, which was then kept at −20 °C for further testing. After the experiment, 6 beef cattle were randomly selected from each treatment group and transported to a commercial slaughterhouse for humane slaughter. Liver tissue samples were immediately collected and stored in liquid nitrogen cryotubes (*n* = 6). After sampling, the samples were transferred to a −80 °C ultra-low temperature freezer for a subsequent analysis.

### 2.4. Parameters of Rumen Fermentation

The rumen fluid pH was assessed using an Orion Star™ A211 benchtop pH meter (Thermo Fisher Scientific, Waltham, MA, USA). The pH meter was calibrated with phosphate buffer solutions of pH 4.00 and 7.00 before use. Measurements were taken at a controlled temperature of 25 °C, and temperature compensation was employed to maintain the precision of the pH readings. The 15 mL centrifuge tubes that contained rumen fluid were subjected to centrifugation at 10,000 revolutions per minute for 10 min, thereby yielding the supernatant. The concentration of ammonia-nitrogen (NH_3_-N) within this supernatant was quantified following the phenol-sodium hypochlorite colorimetric protocol, with measurements taken using a UV1102 spectrophotometer provided by the Techcomp Group (Beijing, China) [19]. The profile of volatile fatty acids (VFAs) in the rumen fluid was analyzed with a GC-6800 gas chromatograph manufactured by the Beibin Tianpu Company (Beijing, China), in accordance with the method described by Broderick and Kang [20].

### 2.5. Nutrient Digestibility

The estimation of apparent nutrient digestibility was executed following the established protocol of the acid-insoluble ash method [21]. The calculation of digestibility of specific nutrients was obtained from the following equation:

$$D_{i}=\{1-[(F_{i}\times T_{A})/(T_{i}\times F_{A})]\}\times 100\%$$

$D_{i}$ represents the apparent digestibility of a given nutrient; $F_{i}$ denotes the concentration of said nutrient in the fecal matter; $F_{A}$ refers to the content of acid-insoluble ash in the feces; $T_{i}$ indicates the nutrient content within the experimental diet; and $T_{A}$ specifies the level of acid-insoluble ash present in the test ration.

### 2.6. Chemical Analysis and Calculation

To determine the DM content of the air-dried TMR and fecal samples, they were oven-dried at 105 °C for 4 h in accordance with AOAC [22] method 930.15. The crude protein (CP) levels in these samples were measured using the combustion technique with a RapidNIII Nitrogen/Protein Analyzer, in accordance with method 999.03 [22]. The neutral detergent fiber (NDF) content was measured with an ANKOM A200i semi-automatic fiber analyzer, according to the method described by Van Soest et al. [23]. For the ash content analysis, a muffle furnace was utilized according to method 975.03 from the AOAC [22]. Calcium (Ca) and phosphorus (P) concentrations were determined using inductively coupled plasma spectroscopy following wet ashing, in accordance with AOAC [22] methods 985.01 A, B, and C. The wet ashing procedure was conducted as described in method 975.03 B(b). The metabolizable energy (ME), net energy for maintenance (NEm), and net energy for gain (NEg) were calculated based on the equations outlined by the NASEM [18].

### 2.7. Serum Indexes

Serum biomarkers, including the total protein (TP), globulin (GLB), albumin (ALB), glucose (GLU), urea nitrogen (UREA), triglycerides (TGs), cholesterol (TC), low-density lipoprotein cholesterol (LDL-C), and high-density lipoprotein cholesterol (HDL-C), were measured using a Hitachi 7600 autoanalyzer, following the manufacturer’s instructions from the Beijing Lidman Biochemical Co., Ltd. (Beijing, China). The enzyme activities of aspartate aminotransferase (AST) and alanine aminotransferase (ALT) were determined according to the International Federation of Clinical Chemistry and Laboratory Medicine guidelines [24], while the alkaline phosphatase (ALP) activity was assessed using the method described by Hitz et al. [25]. Additionally, the hormone-sensitive lipase (HSL), acetyl-CoA carboxylase (ACC), and fatty acid synthase (FAS) levels were quantified with a Multiskan MK3 microplate reader by employing assay kits from the Nanjing Jiancheng Bioengineering Institute (Nanjing, China), as per the provided protocols.

The levels of GAA and creatine were analyzed using an Agilent HPLC1200 chromatographic system (Agilent, Santa Clara, CA, USA), following the methodology described by Owens and Bergen [26]. For the quantification of creatine kinase (CK), creatinine, and adenosine triphosphate (ATP), a Hitachi 7600 automatic biochemistry analyzer (Hitachi, Japan) was employed, adhering to the protocols provided by the Beijing Lidman Biochemistry Co., Ltd. (Beijing, China). Additionally, the serum concentrations of guanidinoacetic acid-N-methyltransferase (GAMT) and arginine–glycine amidinotransferase (AGAT) were assessed using a Multiskan MK3 microplate reader (Thermo Fisher Scientific, USA), following the guidelines detailed in the kits supplied by the Beijing Kangjia Hongyuan Biotechnology Co., Ltd. (Beijing, China).

### 2.8. Antioxidant Indexes in Serum and Liver Tissue

The concentrations of antioxidant indicators in the serum (*n* = 15) and liver tissue (*n* = 6), including the total antioxidant capacity (T-AOC), superoxide dismutase (SOD), catalase (CAT), malondialdehyde (MDA), glutathione (GSH), and glutathione peroxidase (GSH-Px), were measured using a Unico 7200 spectrophotometer (Shanghai Unico Co., Ltd., Shanghai, China). The analysis was performed according to the instructions of the kit provided by the Nanjing Jiancheng Bioengineering Institute (Nanjing, China) and the methods established in previous studies [11,27].

### 2.9. Metabolite Detection and Data Analysis

Liver tissue (50 mg) (*n* = 6) was placed in 2 mL centrifuge tubes with a 6 mm grinding bead. Metabolites were extracted using 400 μL of a 4:1 methanol–water solution, including an internal standard of L-2-chlorophenylalanine at 0.02 mg/mL. The extracted samples were analyzed on a Thermo Fisher Scientific UHPLC-Q Exactive HF-X system for UHPLC-MS/MS, following the conditions outlined in previous work [28,29].

Progenesis QI (Waters Corporation, Milford, MA, USA) was utilized to preprocess the LC-MS raw data, which involved a baseline correction, peak detection, retention time alignment, and peak matching; this ultimately generated a matrix with retention times, mass-to-charge ratios, and intensity values. The dataset underwent a multivariate analysis, including a principal component analysis (PCA) and orthogonal partial least squares discriminant analysis (OPLS-DA), via the ropls package (version 1.6.2) in R to reveal significant patterns. The model reliability was confirmed through a 7-fold cross-validation, and the metabolites with VIP > 1 and *p* < 0.05 were considered significant. These were annotated and mapped to metabolic pathways using the Kyoto Encyclopedia of Genes and Genomes (KEGG) database to highlight the pathways impacted by the metabolite profile shifts.

### 2.10. Transcriptomic Profiling

The transcriptome of 18 liver tissue samples from cattle was profiled using the Illumina NovaSeq 6000 platform. The RNA was extracted from the samples, and its concentration and purity were quantified using a Nanodrop2000 spectrophotometer. The integrity was confirmed by agarose gel electrophoresis, and the RIN values were measured with an Agilent 2100 Bioanalyzer to ensure the sequencing quality. Libraries were prepared using the Illumina TruSeq™ RNA Sample Prep Kit. Paired-end reads were quality-checked with the fastp tool [30], and the cleaned reads were aligned to the reference genome using HISAT2, which employs an orientation-specific algorithm for precise alignment [31]. The transcript assembly was performed with StringTie, a tool designed for the reference-guided reconstruction of transcripts [32]. The gene expression differences between experimental groups were assessed using DESeq2, a statistical method for identifying significant changes in gene expression [33]. The genes that showed a fold change > 1.5 or <0.67 and a *p*-value < 0.05 were deemed differentially expressed. For a functional interpretation, an enrichment analysis of the differentially expressed genes was carried out against the KEGG pathway database to pinpoint overrepresented metabolic pathways. The pathway significance was determined using KOBAS software (3.0 version) by applying the Benjamini–Hochberg correction method to adjust the *p*-values, with a threshold set at 0.05 [34].

### 2.11. Data Statistics and Analysis

Statistical evaluations of growth performance, rumen fermentation parameters, nutrient digestibility, and serum-related indices were conducted using the Mixed procedure within SAS software (version 9.4, SAS Institute Inc., Cary, NC, USA). For this analysis, the fixed effect in the model was the additive treatment (a), while the random effect was attributed to the pen (P). The statistical model used is described as follows:

$$Y_{ij}=\mu +a_{i}+P_{j}+e_{ij}$$

Within this given equation, $Y_{ij}$ signifies the observed value for a specific metric of bulls consuming the *i*-th additive within the *j*-th enclosure. The symbol μ corresponds to the grand mean, $a_{i}$ denotes the influence that the *i*-th additive had on the observed value, and $P_{j}$ reflects the impact from the *j*-th enclosure on this value. $e_{ij}$ is indicative of the random residual associated with each observation.

To investigate the group-wise variations, we utilized Duncan’s post hoc multiple comparisons test. Differences between groups were considered significant if the *p*-value was within the range of 0.01 to 0.05, and highly significant when the *p*-value was below 0.01. At the same time, a, b, and c were used to mark the significant differences between the treatment groups. A trend toward significance, though not statistically confirmed, was indicated by *p*-values that ranged from 0.05 to 0.1.

Spearman correlation analysis was performed in R (version 3.6.3) to assess the relationship between the key differentially expressed genes and metabolites. In the graphical outputs, the significance levels are denoted as follows: * for *p*-values between 0.01 and 0.05, ** for *p*-values between 0.001 and 0.01, and *** for *p*-values less than 0.001. The error bars in the graphs represent the standard error of the mean (SEM).

## 3. Results

### 3.1. Growth Performance, Ruminal Fermentation Parameters, and Nutrient Digestibility

Table 1 presents the impact of the GAA and Met on the growth performance, rumen fermentation, and nutrient digestibility in the beef cattle. The IBW and DMI showed no notable variations across the three groups (*p* > 0.05). In contrast, the groups supplemented with the GAA and GAM demonstrated a marked increase in the ADG and FCE relative to the control group (*p* < 0.05). Additionally, a trend toward an increased FBW was noted in the two feed additive groups, though it did not reach statistical significance (*p* = 0.075).

Regarding ruminal fermentation parameters (Table 2), the GAM group exhibited significantly higher TVFAs and pentanoate concentrations compared with the GAA and CON groups (*p* < 0.05). In contrast, no notable differences were observed in the rumen pH or NH_3_-N levels between the treatment groups.

Regarding the nutrient digestibility (Table 3), the GAM group showed a notable improvement in the digestion of DM and CP compared with the control (*p* < 0.05). Additionally, both the GAA and GAM groups demonstrated markedly enhanced NDF digestibility compared with the control (*p* < 0.01).

### 3.2. Serum Biochemistry, Antioxidant, and Creatine Metabolism Indices

The effects of the GAA and Met supplementation on the serum biochemical profiles of the beef cattle are summarized in Table 4. Both the GAA and GAM groups exhibited significantly higher TP, GLB, and HSL levels than the CON group (*p* < 0.01). Furthermore, the ALB and FAS concentrations were significantly higher in the GAM group than in the control (*p* < 0.05).

Table 5 details the effects on the serum creatine metabolism, revealing that the GAA group had notably elevated GAA levels compared with the control (*p* = 0.027). In contrast, the GAM group demonstrated significantly increased AGAT and GAMT concentrations relative to both the control and GAA groups (*p* < 0.01). Moreover, for AGAT and GAMT, the GAM group exhibited the highest levels, followed by the GAA group, which was still significantly elevated over the CON group (*p* < 0.01).

Table 6 outlines the effects of GAA and Met supplementation on the serum and liver tissue antioxidant parameters in beef cattle. Relative to the control, the serum and liver tissue of beef cattle in the GAA and GAM groups showed significant increases in the T-AOC, SOD, GSH, and CAT levels (*p* < 0.05). In contrast, the MDA concentrations were markedly reduced in these groups (*p* < 0.01).

### 3.3. Metabolite Identification and Sample Relationship Analysis of the Liver

Based on the completion of the raw data preprocessing, the outcomes of the identification statistics pertaining to the total ion count and the number of metabolites are tabulated in Table S2. Using the combined positive and negative ion analysis, 6190 mass spectral peaks were extracted, and 1251 metabolites were finally identified by the search library through the primary and secondary mass spectral data.

As shown in Figure S2, the three treatment groups were well clustered within the group and clearly differentiated between the groups, thus substantiating the reliability of the data.

### 3.4. Differential Metabolite Analysis of the Liver

**Figure 1:**
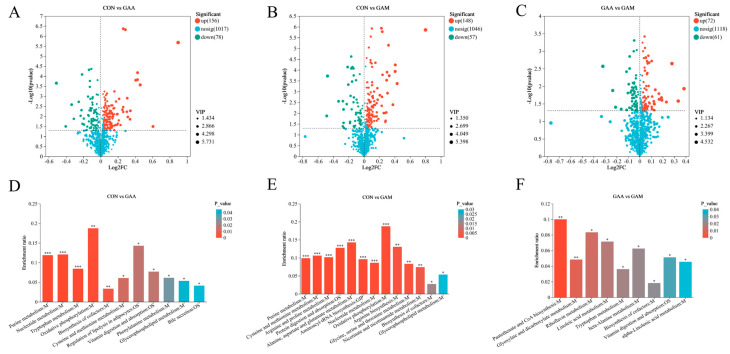
Impacts of guanidinoacetic acid and methionine on liver metabolomics in beef cattle. *n* = 6. (**A**–**C**) Volcano plots illustrating differential metabolites across treatment groups; (**D**–**F**) KEGG pathways enriched by differential metabolites. * for *p*-values between 0.01 and 0.05, ** for *p*-values between 0.001 and 0.01, and *** for *p*-values less than 0.001.

A comprehensive analysis identified a total of 389 metabolites with differential expressions, as detailed in Table S3. The distribution and significance of these changes are illustrated through volcano plots in Figure 1. Comparing the CON group with the GAA group, we observed 234 differentially expressed metabolites: 156 were found to be upregulated, while 78 showed downregulation (Figure 1A). When comparing the CON group against the GAM group, there were 205 differential metabolites identified, with 148 showing increased expression and 37 decreased (Figure 1B). Lastly, the contrast between the GAA and GAM groups highlighted 133 unique metabolites, comprising 72 that were upregulated and 61 that were downregulated (Figure 1C).

### 3.5. KEGG Enrichment Analysis of Differential Metabolites in Liver

Figure 1 illustrates the KEGG functional pathways enriched by differentially expressed metabolites between the treatment groups. In the CON vs. GAA comparison, the key metabolic pathways affected included purine metabolism, tryptophan metabolism, nucleotide metabolism, oxidative phosphorylation, cysteine and methionine metabolisms, and cofactor biosynthesis (Figure 1D). For the CON vs. GAM comparison, the altered metabolites were primarily linked to purine metabolism; arginine and proline metabolisms; cysteine and methionine metabolisms; alanine, aspartate, and glutamate metabolisms; protein digestion and absorption; and aminoacyl-tRNA biosynthesis (Figure 1E). Meanwhile, in the GAA vs. GAM comparison, the enriched pathways included pantothenate and CoA biosynthesis, riboflavin metabolism, linoleic acid metabolism, glycolate and dicarboxylate metabolisms, tryptophan metabolism, and beta-alanine metabolism (Figure 1F).

### 3.6. Key Differential Metabolites in the Liver

Table 7 presents the significant differential metabolites between the treatment groups, with log_2_^FC^ values above zero indicating increased metabolite levels and values below zero reflecting decreased levels. In the GAA group compared with CON, the liver samples exhibited notable elevations in Inosine, LysoPC (20:2(11Z,14Z)/0:0), S-adenosylhomocysteine, PC (17:0/0:0), L-cystine, and ADP, while Choline, 5-hydroxykynurenine, 6-hydroxymelatonin, and NADH were significantly reduced. Relative to CON, the GAM group showed significantly higher concentrations of Inosine, ADP, S-adenosylhomocysteine, L-cystine, Creatine, L-asparagine, L-glutamine, Aspartic acid, L-glutamic acid, LysoPC (20:5(5Z,8Z,11Z,14Z,17Z)/0:0), NAD, and PC (17:0/0:0), whereas the L-serine levels were markedly lower. Additionally, when comparing GAM with GAA, the GAM group demonstrated significantly increased levels of L-kynurenine, Pantothenic acid, N′-formylkynurenine, Carnosine, Stearidonic acid, and Linolenelaidic acid.

### 3.7. Analysis of Gene Expression Level and Sample Relationship in Liver

TPM was employed as a metric for the gene expression levels, with the quantification process carried out using the RSEM software (1.3.3 version). As depicted in Figure S3, the overall gene expression profiles in the livers of beef cattle were comparable across the three experimental groups. The two-dimensional PCA plot portrays the results of assessing the three treatment groups in relation to the mean values of their respective subgroups, revealing a considerable disparity between the three groups and a clear demarcation between them. The three-dimensional PCA plot further highlighted this distinction, showcasing a clearer separation between the CON group and the two feed additive groups, thereby confirming the robustness of the data (Figure S3).

### 3.8. Comparative Gene Expression Analysis in Liver Tissues

**Figure 2:**
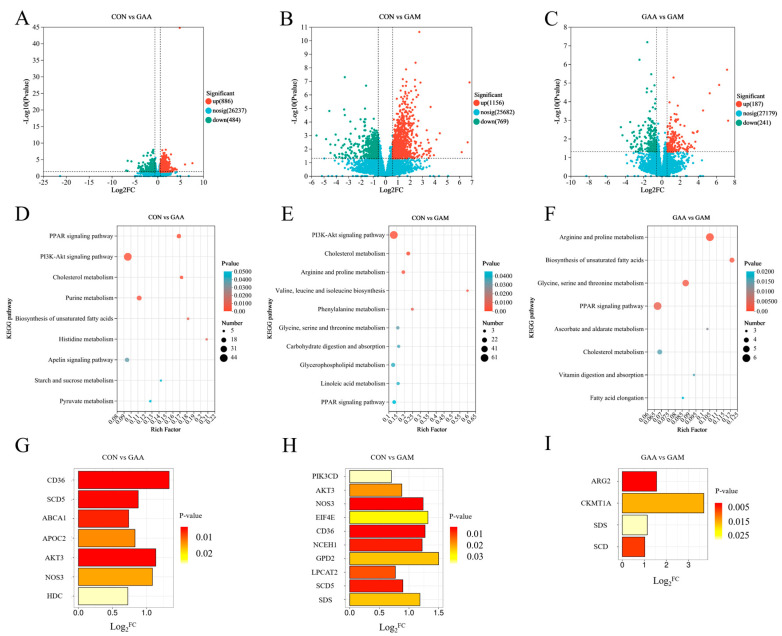
KEGG enrichment pathways and related differentially expressed genes between treatment groups. *n* = 6. (**A**–**C**) Volcano diagrams of differentially expressed genes between groups; (**D**–**F**) KEGG enrichment maps of differentially expressed genes between groups; (**G**–**I**) key differentially expressed genes between groups. Log_2_^FC^ > 0 indicates significant upregulation of genes, while log_2_^FC^ < 0 indicates significant downregulation of genes.

The distribution of the DEGs across the groups is illustrated using volcano plots in Figure 2. As depicted in Figure 2A, a comparison between the liver tissues of beef cattle from the CON and GAA groups revealed a total of 1370 DEGs, with 886 genes showing increased expressions and 484 genes exhibiting decreased expressions in the GAA group. In the comparison between the CON and GAM groups, as depicted in Figure 2B, a total of 1925 DEGs were identified, including 1156 upregulated and 769 downregulated genes in the GAM group. Meanwhile, the contrast between the CON group and GAM group, as shown in Figure 2C, revealed 428 DEGs, where 187 genes exhibited upregulation and 241 showed downregulation in the GAM group relative to the GAA group.

### 3.9. KEGG Enrichment Analysis of Differentially Expressed Genes in Liver

The KEGG pathway enrichment analysis of the DEGs, as illustrated in Figure 2, identified specific metabolic pathways linked to the liver tissue in the beef cattle. In the CON vs. GAA comparison, the key pathways included PPAR signaling, cholesterol metabolism, PI3K–Akt signaling, and histidine metabolism (Figure 2D). When comparing the CON and GAM groups, the enriched pathways encompassed cholesterol metabolism; PI3K–Akt signaling; phenylalanine metabolism; arginine and proline metabolisms; glycine, serine, and threonine metabolism; glycerophospholipid metabolism; and PPAR signaling (Figure 2E). Meanwhile, the GAA vs. GAM contrast highlighted pathways involved in unsaturated fatty acid biosynthesis; arginine and proline metabolisms; glycine, serine, and threonine metabolisms; PPAR signaling; and cholesterol metabolism (Figure 2F).

### 3.10. Key Differentially Expressed Genes in the Liver

As illustrated in Figure 2, the KEGG enrichment analysis of both the DEGs and metabolites highlighted several critical pathways. The key genes associated with PPAR signaling; cholesterol metabolism; PI3K–Akt signaling; histidine metabolism; arginine and proline metabolisms; glycerophospholipid metabolism; and glycine, serine, and threonine metabolisms were identified. The GAA group showed significantly higher expressions of key liver tissue genes, such as *CD36*, *SCD5*, *ABCA1*, *APOC2*, *AKT3*, *NOS3*, and *HDC*, compared with the CON group (Figure 2G). Liver tissues from the GAM group exhibited significantly elevated expressions of genes, such as *PIK3CD*, *AKT3*, *NOS3*, *EIF4E*, *CD36*, *NCEH1*, *GPD2*, *LPCAT2*, *SCD5*, and *SDS*, compared with the CON group (Figure 2H). Furthermore, when comparing the GAM group with the GAA group, the liver tissues from the former showed significantly higher expression levels of *ARG2*, *CKMT1A*, *SDS*, and *SCD* (Figure 2I).

### 3.11. Correlation Between Key Differential Metabolites and Key Differentially Expressed Genes in Liver

**Figure 3:**
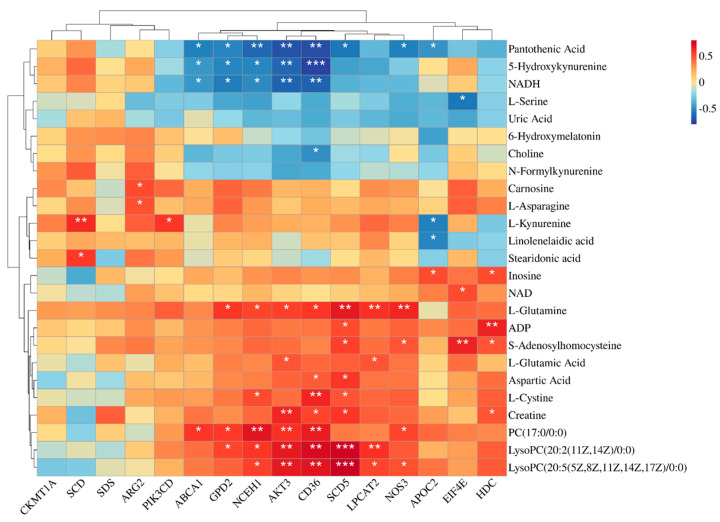
Heat map of the correlations between differentially expressed genes and differential metabolites. * for *p*-values between 0.01 and 0.05, ** for *p*-values between 0.001 and 0.01, and *** for *p*-values less than 0.001. *n* = 6.

Figure 3 illustrates the results of a correlation analysis that investigated the relationships between the key differential metabolites and the DEGs. Notably, *AKT3* exhibited significant positive correlations with several lipid metabolism-related metabolites, including PC (17:0/0:0), LysoPC (20:2(11Z,14Z)/0:0), and LysoPC (20:5(5Z,8Z,11Z,14Z,17Z)/0:0), as well as protein-metabolism-related compounds, such as Creatine, L-glutamic acid, and L-glutamine. Similarly, genes like *GPD2*, *NCEH1*, *CD36*, *SCD5*, and *LPCAT2* mirrored this trend, where they showed significant positive correlations with the same metabolites as *AKT3*. Moreover, *NOS3* displayed significant positive associations specifically with LysoPC (20:5(5Z,8Z,11Z,14Z,17Z)/0:0), S-adenosylhomocysteine, PC (17:0/0:0), and L-glutamine.

## 4. Discussion

In this study, incorporating GAA into the diet of the beef cattle was found to significantly boost the growth performance, a result that corroborates previous findings [11,17,35]. Li et al. [35] found that the ADG in Angus bulls significantly improved with increasing GAA supplementation of 0.3, 0.6, and 0.9 g/kg dry matter. Meanwhile, Yi et al. [11] noted that the ADG in Angus steers improved with GAA supplementation at 0.8 g/kg and 1.6 g/kg, but they also observed that beyond a certain concentration, further increases in GAA did not lead to additional improvements in the ADG. The possibility arises that this phenomenon stems from elevated GAA supplementation triggering an increased methyl demand in the body, rendering the methyl yield from dietary breakdown inadequate to accommodate the augmented GAA provision. In this study, the IBW remained consistent across all treatment groups, while the GAM group exhibited the highest FBW, ADG, and FCE, surpassing the GAA group by 2.24%, 6.9%, and 10%, respectively. These results indicate that adding Met further improved the growth performance of the beef cattle. However, unlike the findings of Liu et al. [17], GAA supplementation in this study did not lead to an increased feed intake. Furthermore, Li et al. [36] reported that GAA addition significantly raised the DMI of Jinjiang bulls, which is also discordant with the findings in this study. This discrepancy might be attributed to variations in the breed and dietary composition between the studied beef cattle populations.

Rumen fermentation parameters reflect rumen health, and in this study, the rumen pH showed no significant variation between the groups, remaining stable at a healthy level. This suggests that neither the GAA nor Met negatively affected the ruminal health. Pertinent literature has revealed that GAA supplementation can lead to increased ruminal TVFA concentrations in beef cattle [17,35], a finding that aligns with the outcomes of our investigation. Since TVFAs originate from the degradation of nutrients by rumen microorganisms, elevated TVFAs may imply altered digestibility [37]. Furthermore, the significantly elevated concentration of valeric acid in the rumen implies that the addition of GAA and Met potentially modulated the ruminal fermentation environment and influenced the breakdown of nutrients by rumen microbial populations. This observation was supported by the results of the apparent nutrient digestibility assessment. Our study revealed a significant increase in the NDF digestibility in the GAA and GAM groups relative to the CON group. Moreover, the DM and CP digestibilities in the GAM group exceeded those of the CON group, consistent with prior research [11,12,17,35]. The increase in nutrient digestibility also explains the significantly higher ADG in the absence of a difference in the DMI. This suggests that including GAA and Met in the diet could positively impact nutrient digestion in beef cattle.

The TP, including ALB and GLB, is an important indicator for detecting the synthetic function of the liver [38]. ALB plays a vital role in preserving blood osmolarity and ensuring nutrient provision [39], while GLB plays an immunological role in animals [40]. In this study, GAA supplementation led to elevated serum concentrations of TP and GLB, with the ALB levels notably higher in the GAM group than in the CON group. These findings are consistent with those documented by Li et al. [36]. In this study, TP, ALB, and GLB all experienced alterations within the standard concentration range, whereas ALT, AST, and ALP are pivotal markers for determining the normalcy or otherwise of liver function [41]. The observation that these latter enzymes did not display significant disparities between the three treatment groups substantiates that the increases in the TP, ALB, and GLB were not a consequence of abnormal liver function. UREA is a product of protein metabolism [42], which was increased in the serum of the GAM group. Collectively, these findings imply that the inclusion of GAA and Met may have moderately enhanced the liver’s protein-synthesizing function, thereby fostering improved protein metabolism within the organism. HSL, which is associated with lipolysis metabolism [43], was significantly elevated in the sera of both the GAA and GAM groups. Similarly, FAS, which is linked to fatty acid synthesis [44], also showed a significant increase in the serum of the GAM group. The possible reason for this phenomenon is the bidirectional regulation of energy metabolism. Specifically, the addition of GAA and Met may increase the body’s energy supply, which may stimulate adipose tissue to store fatty acids (FAS increase); in addition, GAA and Met may improve the body’s energy utilization, which increases HSL and promotes fat decomposition. Together, these observations suggest that GAA and Met supplementation may have an effect on the lipid metabolism in beef cattle. However, the serum lipid indices (TC, TG, HDL-C, and LDL-C) failed to reveal significant discrepancies between the three treatment groups, potentially due to the organism’s inherent regulatory mechanisms governing lipid metabolism [45].

In this study, the serum GAA levels were significantly higher in the GAA group compared with the CON group, with a similar pattern observed in the GAM group. These results align with previous findings reported by Yi et al. [11]. The results suggest that not all dietary GAA was degraded by rumen microorganisms; instead, a portion successfully entered the bloodstream, allowing it to fulfill its intended function. The lesser increase in the serum GAA concentration observed in the beef cattle from the GAM group may be attributed to the heightened utilization of GAA by the body resulting from Met supplementation. The concentrations of AGAT and GAMT, which are key rate-limiting enzymes involved in GAA synthesis and creatine metabolism [46], were significantly higher in both the GAA and GAM groups compared with the control. This implies that the GAA supplementation stimulated creatine metabolism and augmented the demand for GAA within the animals’ bodies. In this study, no significant differences were observed in the serum creatine, creatine kinase, or ATP levels across the three groups. This could have been due to the elevated demand for creatine in the muscle tissues of the beef cattle, aligning with the higher ADG recorded. Another possible reason for this consistency is that the addition of GAA improves the body’s energy utilization, which leads to no significant increase in ATP in the blood. In addition, the animal’s own regulatory effect may also be the reason why there were no significant differences in the above three indicators [47].

Creatine is known to possess the capacity to neutralize oxygen free radicals [48]; hence, GAA supplementation may exert an indirect antioxidative effect. Previous research has demonstrated the beneficial effects of GAA on enhancing the antioxidant status in animals. For example, Li et al. [12] observed that supplementing diets with GAA increased the antioxidant capacity in both the rumens and sera of lambs. Consistent with these findings, Yi et al. [11] reported increased CAT concentrations and reduced MDA levels in the sera of Angus steers following GAA supplementation. These results align with the observations from the present research. T-AOC represents the overall levels of antioxidants, enzymes, and other components involved in oxidative defense within an animal’s body. It embodies the comprehensive antioxidant level derived from diverse antioxidant substances and antioxidant enzymes present in the animal system [49]. Typically, T-AOC remains within a physiological range and can be influenced by diet, exercise, and metabolic conditions. In our study, despite the animals being healthy, the supplementation with GAA and Met significantly increased the T-AOC levels in both the serum and liver tissues. This suggests that GAA supplementation not only provides metabolic benefits but also enhances antioxidant defense mechanisms. One possible explanation for this significant increase in T-AOC is the upregulation of antioxidant enzyme activity, particularly SOD and CAT, which play distinct roles in mitigating oxidative stress. SOD converts superoxide anion radicals (O_2_⁻) into hydrogen peroxide and oxygen, while CAT further decomposes hydrogen peroxide into water and oxygen [50,51]. The elevated activities of these enzymes in both serum and liver tissues indicate an enhanced endogenous antioxidant response. Notably, the increase in CAT activity suggests a potential metabolic adaptation to counteract oxidative stress associated with increased metabolic rates due to GAA supplementation. This supports the notion that while GAA promotes growth, it may also lead to increased metabolic activity, potentially inducing mild oxidative stress, which is then neutralized through an upregulated antioxidant defense system. Moreover, the reduction in MDA, a byproduct of lipid peroxidation [52], further supports the hypothesis that GAA supplementation improves the body’s ability to manage oxidative stress. The observed increase in GSH levels in serum and liver tissues, without a corresponding significant increase in the GSH-Px activity, may be attributed to the increased consumption of GSH-Px in response to oxidative stress, resulting in a higher GSH turnover [53]. This reflects a compensatory mechanism where the body maintains redox homeostasis through increased antioxidant production rather than solely relying on enzymatic activity. A substantial proportion of nutrients assimilated by the stomach and intestines are conveyed via the bloodstream to the liver, where they undergo metabolic transformations [54] before being distributed to various tissues and organs throughout the body via the circulatory system. The consistency of the changes in the above antioxidant indicators in serum and liver tissues leads to the view that changes in liver metabolic function may affect the corresponding metabolic indicators in the blood [55]. In addition, this study also observed significant changes in the levels of serum indicators related to protein metabolism and lipid metabolism to varying degrees. Hence, the metabolomic analysis of liver tissues in this study could facilitate the exploration of the effects of GAA supplementation on liver metabolic functions, while transcriptomic analysis could elucidate the precise mechanisms underlying metabolic function changes at the gene expression level.

Relative to the CON group, the concentrations of Inosine, ADP, S-adenosylhomocysteine, L-cysteine, and PC (17:0/0:0) were elevated in both the GAA group and GAM group. Inosine is a key intermediate in purine metabolism and also participates in nucleotide metabolism and energy metabolism [56]; ADP is also involved in the above metabolism and is also a marker of oxidative phosphorylation in the organism [57]. The increased levels observed in both the GAA and GAM groups indicate that GAA supplementation enhances energy metabolism in beef cattle, providing abundant substrates for the synthesis of amino acids, sugars, nucleic acids, and fatty acids. S-adenosylhomocysteine (SAH), a crucial metabolite in creatine synthesis, is generated during the conversion of S-adenosylmethionine to creatine with GAA [58]. Elevated SAH levels indicate increased creatine production in the hepatic tissues of both the GAA and GAM groups; in addition, SAH is metabolized to homocysteine, which reacts with betaine to be able to produce methionine, while choline is a precursor substance of betaine [59]. The lower Choline levels observed in the GAA group, relative to the control, may indicate a higher requirement for betaine, indirectly suggesting that GAA supplementation enhanced the hepatic creatine metabolism. Furthermore, hepatic creatine levels in the GAM group were markedly elevated relative to the control, indicating that Met supplementation further enhanced the creatine synthesis in the liver. This suggests that the addition of Met further enhanced the creatine synthesis in the liver of the beef cattle. These changes corresponded to the changes in serum indicators of creatine metabolism. PC (17:0/0:0), LysoPC (20:2(11Z,14Z)/0:0), and LysoPC (20:5(5Z,8Z,11Z,14Z,17Z)/0:0) were all enriched in the glycerophospholipid metabolic pathway, which has the roles of constructing cell membranes, participating in lipid metabolism, and regulating the cholesterol metabolism in animals [60]. The differing degrees of increase in the content of these three substances in the GAA group and GAM group indicate that the GAA supplementation stimulated the glycerophospholipid metabolic pathway, and the augmentation of this pathway suggests potential enhancement of lipid metabolism within the livers of the beef cattle. This finding also provides an explanation for the increased serum lipase content observed.

Previous research has demonstrated the impact of GAA supplementation on amino acid metabolism in animals [10], which aligns with the results observed in the current investigation. In the hepatic tissues of the GAA group, the concentrations of 5-hydroxykynurenine and 6-hydroxymelatonin, which are both associated with the tryptophan metabolic pathway, were significantly reduced relative to the control. Tryptophan, an essential amino acid, is primarily used by the host for protein synthesis [61]. The decreased levels of its related metabolites suggest that GAA supplementation suppresses tryptophan catabolism, thereby supporting improved protein synthesis. L-cystine, which is known for supporting hepatic detoxification [62], significantly increased in both the GAA and GAM groups, suggesting that GAA supplementation boosts the liver detoxification capacity. Notably, Met supplementation significantly elevated the Aspartic acid, L-asparagine, L-glutamic acid, and L-glutamine levels while reducing L-serine in the liver of the GAM group cattle. These amino acids are integral to processes such as protein digestion and absorption. These alterations indicate increased activity in essential metabolic pathways related to protein synthesis, such as aminoacyl-tRNA biosynthesis; arginine and proline metabolisms; cysteine and methionine metabolisms; glycine, serine, and threonine metabolisms; and alanine, aspartate, and glutamate metabolisms. The rise in amino acid metabolites in the GAM group suggests that Met supplementation enhanced the hepatic amino acid metabolism. This reinforces the idea that beef cattle in the GAM group exhibited enhanced protein synthesis capacity, aligning with their greater daily weight gain.

NADH is known as the reduced coenzyme I and is involved in glycolysis and cellular respiration [63], while NADPH is known as the reduced coenzyme II and plays energy supply, signal transduction, and antioxidant roles in cells [64]. Although NADPH is widely considered to be the main reducing equivalent involved in the antioxidant defense system (especially through its role in the glutathione reductase and thioredoxin systems), recent studies have also emphasized that NADH has certain antioxidant properties [65]. NADH can directly scavenge some reactive oxygen species (ROS) through its inherent reducing ability and can indirectly promote antioxidant regeneration, although to a lesser extent than NADPH; in addition, fluctuations in NADH/NAD⁺ levels can reflect the cellular redox status and metabolic activities, especially those within mitochondria [63]. Therefore, the significant decrease in NADH levels observed in the GAA group and the significant increase in the level of its oxidized counterpart NAD+ in the GAM group in this study not only reflect changes in the energy metabolism but also partially contribute to redox regulation in liver tissue. Unlike NADH, NADPH is primarily produced through the pentose phosphate pathway (PPP), a key metabolic pathway that runs in parallel with glycolysis. The PPP plays a crucial role in cellular antioxidant defense by providing NADPH, which is essential for maintaining the reduced state of glutathione and supporting other antioxidant systems [64]. Although NADPH was not specifically detected in our metabolomics analysis, its indirect role in redox homeostasis is well recognized. The enhanced antioxidant enzyme activities observed in the GAA and GAM groups may also reflect upstream activation of the metabolic pathways, including the PPP, which deserves further investigation in future studies. Additionally, hepatic concentrations of Pantothenic Acid and Carnosine, which are critical for Pantothenate and CoA biosynthesis and beta-Alanine metabolism, respectively, were markedly elevated in the GAM group relative to the GAA group. Pantothenic Acid is known to contribute to cellular energy metabolism, substance synthesis, and regulation [66], whereas Carnosine is intimately tied to skeletal muscle energy metabolism and protein synthesis [67]. Furthermore, L-Kynurenine and N′-Formylkynurenine, which are associated with tryptophan metabolism, as well as Stearidonic acid and Linolenelaidic acid, which are linked to α-linolenic acid metabolism, showed significant increases in the hepatic tissues of the GAM group. The changes in these metabolites suggest that protein and lipid metabolisms were more active in the livers of beef cattle in the GAM group than in the GAA group. This finding corresponds to the observed higher FBW and ADG in the GAM group.

To explore the mechanisms underlying the observed metabolite changes, a transcriptomic analysis of liver tissues was conducted. The PI3K–AKT signaling pathway, which is known to regulate energy metabolism and protein synthesis in animals [68,69], was identified as a key contributor. In this pathway, the *AKT3* gene was significantly upregulated in the liver of beef cattle from both the GAA group and GAM group, while the *PIK3CD* gene showed significant upregulation, specifically in the GAM group. This result indicates that the PI3K–AKT pathway was activated in both the GAA and GAM groups, suggesting that GAA supplementation promoted improved energy and protein metabolism in the hepatic tissues of beef cattle. This was flanked by the results showing that *AKT3* had significant positive correlations with Creatine, L-glutamic acid, and L-glutamine. *NOS3* is a downstream target gene of the PI3K–AKT pathway that is responsible for the transcriptional regulation of *eNOS* production. *eNOS*, in turn, catalyzes the conversion of L-arginine and reactive oxygen species (O_2_) into L-citrulline and nitric oxide (NO), a process that aids in the elimination of reactive oxygen species from the body [70]. At the same time, NO helps to dilate blood vessels so that more blood flows through the liver [71], which can bring more nutrients to the liver for conversion and metabolism. The significant upregulation of *NOS3* in the liver tissues of beef cattle from both the GAA group and GAM group indicates that GAA supplementation can elevate *NOS3* expression by activating the PI3K–AKT pathway, thereby contributing to the improvement of the liver’s antioxidant capacity and the enhancement of the overall nutrient metabolism in beef cattle. The positive correlations between *NOS3* and phospholipids, creatine metabolism-related metabolites, and L-glutamine were also confirmed. The gene *EIF4E*, which plays a key role downstream in the PI3K–AKT signaling pathway and is essential for regulating protein synthesis [72], was significantly upregulated in the GAM group compared with the control group. These findings imply that the simultaneous administration of GAA and Met could improve hepatic protein synthesis in beef cattle. Moreover, this study observed notable changes in genes associated with amino acid metabolism. For example, *HDC* was notably elevated in the GAA group, where it contributed to histidine metabolism by converting histidine into histamine [73]. In contrast, *SDS*, which is involved in serine metabolism by facilitating the conversion of serine to pyruvate [74], exhibited significant upregulation, specifically in the GAM group. The gene expression patterns suggest that GAA supplementation enhances amino acid metabolism in the liver, and the addition of Met may further increase the protein synthesis efficiency.

Significant enrichment of both the PPAR signaling pathway and cholesterol metabolism was observed in the GAA and GAM groups. Central to the PPAR pathway are genes like *CD36* and *SCD5*. *CD36* acts as a key regulator of fatty acid sensing and plays a pivotal role in modulating lipid metabolism [75], while *SCD5* is essential as a rate-limiting enzyme for monounsaturated fatty acid production. Specifically, *SCD5* facilitates the conversion of stearoyl coenzyme A (C18:0) and palmitoyl coenzyme A (C16:0) into oleic acid (C18:1) and palmitoleic acid (C16:1), respectively [76]. In the current research, both *CD36* and *SCD5* exhibited significant upregulation in the GAA and GAM groups, suggesting heightened lipid metabolic activity. These genes were also associated with specific lipid-related metabolites, including LysoPC (20:2(11Z,14Z)/0:0) and LysoPC (20:5(5Z,8Z,11Z,14Z,17Z)/0:0). These metabolites exhibited a significant positive correlation with one another. These findings collectively suggest that GAA supplementation effectively regulates hepatic lipid metabolism. In terms of cholesterol metabolism, *ABCA1* and *APOC2* were notably upregulated in the GAA group, whereas *NCEH1* showed significant upregulation in the GAM group. Additionally, genes involved in glycerophospholipid metabolism, such as *GPD2* and *LPCAT2*, were significantly upregulated in the GAM group. These gene expression changes further support the regulatory role of GAA supplementation on lipid metabolism in beef cattle livers. Additionally, the expression of *SCD*, a gene linked to the PPAR pathway, was notably higher in the GAM group compared with the GAA group, highlighting the added effect of Met supplementation. Analogously, *ARG2* and *CKMT1A*, genes associated with arginine and proline metabolism, along with *SDS*, a gene involved in serine metabolism, all displayed significant upregulation in the GAM group. These gene expression alterations collectively suggest that Met supplementation can further potentiate lipid metabolism and amino acid metabolism in the livers of beef cattle. Furthermore, additional transcriptomic and metabolomic analyses of the longissimus lumborum (LL) muscle revealed that dietary GAA supplementation improved the meat quality, antioxidant capacity, and modulated nutritional and fatty acid profiles. The addition of RPM further enhanced the antioxidant capacity and altered the fatty acid composition. These findings were associated with significant changes in genes and metabolites in amino acid and lipid metabolic pathways [27]. Thus, GAA and RPM not only affect liver metabolism but also have meaningful effects on muscle metabolism, supporting improved growth performance and meat quality. Furthermore, given that beef is ultimately intended for human consumption, it is important to evaluate not only the effects of dietary interventions on animal performance and metabolism but also their potential impacts on the meat quality and food safety. Although the amount of methionine supplementation in this study was within the nutritional recommendations and physiological suitability for ruminants, the long-term consequences of such interventions—particularly the accumulation of amino acids or related metabolites in edible tissues—need careful consideration. Although no adverse effects on the liver function were observed in the current trial and improvements in the antioxidant capacity and growth performance were evident, further studies are needed to assess the nutritional safety of methionine-enriched meat products for human consumption. Future research should combine comprehensive food safety assessments with nutritional assessments relevant to humans to ensure that dietary supplementation strategies for livestock not only benefit production efficiency but also comply with public health considerations.

## 5. Conclusions

In summary, incorporating GAA into the diet enhanced the growth performance, nutrient digestibility, and serum protein and creatine metabolisms of beef cattle, while also boosting their antioxidant capacity. Furthermore, the inclusion of Met further augmented the nutrient digestibility. Furthermore, it was found that incorporating GAA into the diet led to the upregulation of metabolites involved in energy and protein and lipid metabolisms within the liver tissues. The Met supplementation, on the other hand, moderately enhanced the amino acid metabolism in the livers of the beef cattle. The changes observed in these metabolites were closely linked to the increased expression of genes that regulate protein and lipid metabolic processes. Therefore, the results suggest that combining GAA with methyl donors like Met could be beneficial for the rearing of beef cattle. This study provides valuable insights for optimizing the use of GAA and Met in beef cattle production, highlighting their potential to improve the metabolic efficiency and overall animal performance during the rearing process.

## Figures and Tables

**Table 1:** Effect of dietary addition of guanidinoacetic acid and methionine on growth performance in beef cattle.

Items ^1^	Treatment Groups			SEM	*p*-Value
	CON	GAA	GAM		
IBW (kg)	453.96	452.43	453.87	4.43	0.988
FBW (kg)	594.00	614.14	627.87	6.18	0.075
ADG (kg)	1.00 ^b^	1.16 ^a^	1.24 ^a^	0.03	0.002
DMI (kg)	11.20	11.42	11.43	0.13	0.735
FCE	0.09 ^b^	0.10 ^a^	0.11 ^a^	0.003	0.004

^1^ IBW: initial body weight; FBW: final body weight; ADG: average daily gain; DMI: daily dry matter intake; FCE: feed conversion efficiency. a and b were used to mark the significant differences between the treatment groups. *n* = 15.

**Table 2:** Effect of dietary addition of guanidinoacetic acid and methionine on ruminal fermentation parameters in beef cattle.

Items ^1^	Treatment Groups			SEM	*p*-Value
	CON	GAA	GAM		
pH	6.57	6.44	6.42	0.035	0.153
NH_3_-N (mg/100 mL)	10.75	9.32	9.53	0.516	0.506
TVFAs (mmol/L)	79.92 ^b^	92.51 ^ab^	101.18 ^a^	3.38	0.023
Acetate %	70.78	71.17	70.87	0.002	0.647
Propionate %	16.61	16.29	16.31	0.002	0.800
Isobutyrate %	0.96	0.91	0.91	0.001	0.707
Butyrate %	9.71	9.73	9.97	0.002	0.857
Isopentanoate %	1.49	1.39	1.37	0.001	0.778
Pentanoate %	0.45 ^b^	0.50 ^ab^	0.57 ^a^	0.0002	0.013
Acetate/propionate	4.28	4.38	4.36	0.059	0.781

^1^ NH_3_-N: ammonia-nitrogen; TVFAs: total volatile fatty acids. a and b were used to mark the significant differences between the treatment groups. *n* = 15.

**Table 3:** Effect of dietary addition of guanidinoacetic acid and methionine on nutrient digestibility in beef cattle.

Items ^1^	Treatment Groups			SEM	*p*-Value
	CON	GAA	GAM		
DM %	60.90 ^b^	64.44 ^ab^	66.57 ^a^	0.009	0.034
CP %	52.20 ^b^	56.09 ^ab^	60.29 ^a^	0.012	0.011
NDF %	52.12 ^b^	60.06 ^a^	63.36 ^a^	0.016	0.010

^1^ DM: dry matter; CP: crude protein; NDF: neutral detergent fiber. a and b were used to mark the significant differences between the treatment groups. *n* = 15.

**Table 4:** Effects of guanidinoacetic acid and methionine on serum biochemical indices in beef cattle.

Items ^1^	Treatment Groups			SEM	*p*-Value
	CON	GAA	GAM		
TP (g/L)	71.41 ^b^	76.58 ^a^	78.53 ^a^	0.82	<0.001
ALB (g/L)	30.75 ^b^	31.68 ^ab^	32.29 ^a^	0.22	0.013
GLB (g/L)	40.66 ^b^	44.90 ^a^	46.23 ^a^	0.71	0.002
UREA (mmol/L)	1.99 ^b^	2.18 ^ab^	2.37 ^a^	0.06	0.021
GLU (mmol/L)	3.81	3.56	3.78	0.11	0.632
TC (mmol/L)	3.44	3.19	3.44	0.10	0.504
TGs (mmol/L)	0.17	0.17	0.20	0.01	0.187
HDL-C (mmol/L)	2.01	1.89	2.03	0.05	0.442
LDL-C (mmol/L)	1.11	0.93	1.13	0.06	0.267
ALT (U/L)	35.09	30.71	42.13	2.22	0.101
AST (U/L)	101.01	98.84	101.19	2.85	0.936
ALP (U/L)	150.76	139.36	160.81	6.67	0.428
ACC (ng/mL)	1.81	2.07	2.18	0.07	0.114
HSL (ng/mL)	10.52 ^b^	14.11 ^a^	14.59 ^a^	0.34	<0.001
FAS (ng/mL)	8.13 ^b^	8.57 ^ab^	9.30 ^a^	0.20	0.043

^1^ TP: total protein, ALB: albumin, GLB: globulin, UREA: urea nitrogen, GLU: glucose, TC: cholesterol, TGs: triglycerides, HDL-C: high-density lipoprotein cholesterol, LDL-C: low-density lipoprotein cholesterol, ALT: alanine aminotransferase, AST: aspartate aminotransferase, ALP: alkaline phosphatase, ACC: acetyl-CoA carboxylase, HSL: hormone-sensitive esterase, FAS: fatty acid synthase. a and b were used to mark the significant differences between the treatment groups. *n* = 15.

**Table 5:** Effect of guanidinoacetic acid and methionine on serum creatine metabolism indexes in beef cattle.

Items ^1^	Treatment Groups			SEM	*p*-Value
	CON	GAA	GAM		
GAA (mg/L)	44.64 ^b^	74.35 ^a^	61.70 ^ab^	4.54	0.027
AGAT (U/L)	22.62 ^c^	27.11 ^b^	30.48 ^a^	0.80	<0.001
GAMT (U/L)	270.59 ^c^	332.53 ^b^	370.15 ^a^	8.97	<0.001
Creatine (mg/L)	3.27	3.25	3.65	0.23	0.733
CK (U/L)	381.99	316.04	337.10	25.22	0.577
ATP (mg/L)	8.98	10.23	11.28	0.57	0.256
CREA (μmol/L)	140.69	145.01	147.85	3.29	0.680

^1^ GAA: guanidinoacetic acid, AGAT: arginine–glycine amidinotransferase, GAMT: guanidinoacetic acid-N-methyltransferase, CK: creatine kinase, ATP: adenosine triphosphate, CREA: creatinine. a, b and c were used to mark the significant differences between the treatment groups. *n* = 15.

**Table 6:** Effects of guanidinoacetic acid and methionine on serum and liver tissue antioxidant indices in beef cattle.

Items ^1^	Treatment Groups			SEM	*p*-Value
	CON	GAA	GAM		
Serum antioxidant indices					
T-AOC (mmol/L)	0.42 ^b^	0.48 ^a^	0.47 ^a^	0.01	<0.001
SOD (U/mL)	147.88 ^b^	163.32 ^a^	161.12 ^a^	1.49	<0.001
GSH (μmol/L)	15.11 ^b^	17.54 ^a^	17.66 ^a^	0.32	<0.001
GSH-Px (U/mL)	134.03	149.42	155.55	4.44	0.124
CAT (U/mL)	2.54 ^b^	4.27 ^a^	3.88 ^a^	0.15	<0.001
MDA (nmol/mL)	4.96 ^a^	3.94 ^b^	3.95 ^b^	0.13	<0.001
Liver tissue antioxidant indices					
T-AOC (mmol/g)	0.55 ^b^	0.74 ^a^	0.78 ^a^	0.04	0.014
SOD (U/mg)	223.89 ^b^	315.95 ^a^	310.09 ^a^	15.20	0.011
GSH (µmol/g)	17.12 ^b^	18.96 ^a^	21.78 ^a^	0.67	0.007
GSH-Px (U/mg)	136.69	153.29	161.03	5.95	0.244
CAT (U/mg)	2.57 ^b^	4.73 ^a^	4.08 ^a^	0.34	0.020
MDA (nmol/mg)	5.66 ^a^	3.73 ^b^	3.71 ^b^	0.07	0.007

^1^ T-AOC: total antioxidant capacity, SOD: superoxide dismutase, GSH: glutathione, GSH-Px: glutathione peroxidase, CAT: catalase, MDA: malondialdehyde. a and b were used to mark the significant differences between the treatment groups.

**Table 7:** Key differential metabolites between groups.

Metabolite	VIP	FC	*p*	Type
CON vs. GAA				
Inosine	2.060	1.074	0.006	Up
ADP	1.511	1.038	0.007	Up
S-adenosylhomocysteine	1.033	1.023	0.021	Up
L-cystine	1.834	1.066	0.007	Up
Choline	1.060	0.983	0.019	Down
5-hydroxykynurenine	2.018	0.925	0.005	Down
6-hydroxymelatonin	1.215	0.964	0.036	Down
LysoPC (20:2(11Z,14Z)/0:0)	2.105	1.081	0.040	Up
PC (17:0/0:0)	1.640	1.039	0.001	Up
NADH	1.373	0.9594	0.028	Down
CON vs. GAM				
Inosine	1.744	1.067	0.024	Up
ADP	1.752	1.049	0.003	Up
S-adenosylhomocysteine	1.304	1.030	0.008	Up
L-cystine	1.699	1.062	0.032	Up
Creatine	1.136	1.023	0.041	Up
L-asparagine	1.009	1.021	0.027	Up
Aspartic acid	1.254	1.026	0.024	Up
L-glutamine	1.590	1.037	0.001	Up
L-glutamic Acid	1.070	1.019	0.041	Up
L-serine	1.041	0.977	0.014	Down
LysoPC(20:5(5Z,8Z,11Z,14Z,17Z)/0:0)	2.074	1.085	0.005	Up
PC(17:0/0:0)	1.452	1.031	0.004	Up
NAD	1.085	1.024	0.045	Up
GAA vs. GAM				
L-kynurenine	4.532	1.214	0.002	Up
Pantothenic acid	1.057	1.011	0.028	Up
N′-formylkynurenine	3.154	1.140	0.021	Up
Carnosine	1.736	1.030	0.039	Up
Stearidonic acid	1.384	1.020	0.021	Up
Linolenelaidic acid	2.495	1.091	0.048	Up

CON vs. GAA: FC (GAA/CON); CON vs. GAM: FC (GAM/CON); GAA vs. GAM: FC (GAM/GAA); FC > 1 indicates upregulation of metabolites and FC < 1 indicates downregulation of metabolites. *n* = 6. VIP means variable importance in projection; FC means fold change.

## Data Availability

Data will be provided upon reasonable request.

## Supplementary Materials

The following supporting information can be downloaded from https://www.mdpi.com/article/10.3390/antiox14050559/s1. Table S1: Diet formula and nutritional composition during the trial period; Table S2: Liver metabolome total ion count and metabolite count statistics; Table S3: Liver metabolite details; Figure S1: Rumen degradation rate and intestinal release rate of guanidinoacetic acid and methionine; Figure S2: Sample relationship analysis plot for the liver metabolome; Figure S3: Gene expression distribution, with two-dimensional and three-dimensional principal component analysis plots of the liver transcriptome.

## Abbreviations

The following abbreviations are used in this manuscript:

GAA	Guanidinoacetic acid
Met	Methionine
ADG	Average daily gain
FCE	Feed conversion efficiency
NDF	Neutral detergent fiber
TVFAs	Total volatile fatty acids
DM	Dry matter
CP	Crude protein
TMR	Total mixed ration
IBW	Initial body weight
FBW	Final body weight
DMI	Dry matter intake
NH3-N	Ammonia-nitrogen
VFAs	Volatile fatty acids
Calcium	Ca
Phosphorus	P
ME	Metabolizable energy
NEm	Net energy for maintenance
NEg	Net energy for gain
TP	Total protein
GLB	Globulin
ALB	Albumin
GLU	Glucose
UREA	Urea nitrogen
TGs	Triglycerides
TC	Cholesterol
LDL-C	Low-density lipoprotein cholesterol
HDL-C	High-density lipoprotein cholesterol
ALT	Alanine aminotransferase
AST	Aspartate aminotransferase
ALP	Alkaline phosphatase
HSL	Hormone-sensitive lipase
ACC	Acetyl-CoA carboxylase
FAS	Fatty acid synthase
CK	Creatine kinase
ATP	Adenosine triphosphate
GAMT	Guanidinoacetic acid-N-methyltransferase
AGAT	Arginine–glycine amidinotransferase
T-AOC	Total antioxidant capacity
SOD	Superoxide dismutase
CAT	Catalase
MDA	Malondialdehyde
GSH	Glutathione
GSH-Px	Glutathione peroxidase
PCA	Principal component analysis
OPLS-DA	Orthogonal partial least squares discriminant analysis
KEGG	Kyoto Encyclopedia of Genes and Genomes
