# Supplementary material for: Guanidinoacetic Acid and Methionine Supplementation Improve the Growth Performance of Beef Cattle via Regulating the Antioxidant Levels and Protein and Lipid Metabolisms in Serum and Liver
Source: Antioxidants (Basel). 2025 May 8;14(5):559. doi: 10.3390/antiox14050559 (PMC12108366; doi:10.3390/antiox14050559)
Supplement: Supplementary file 1 [file antioxidants-14-00559-s001.zip › antioxidants-3513798-supplementary/Figure S2.pdf]

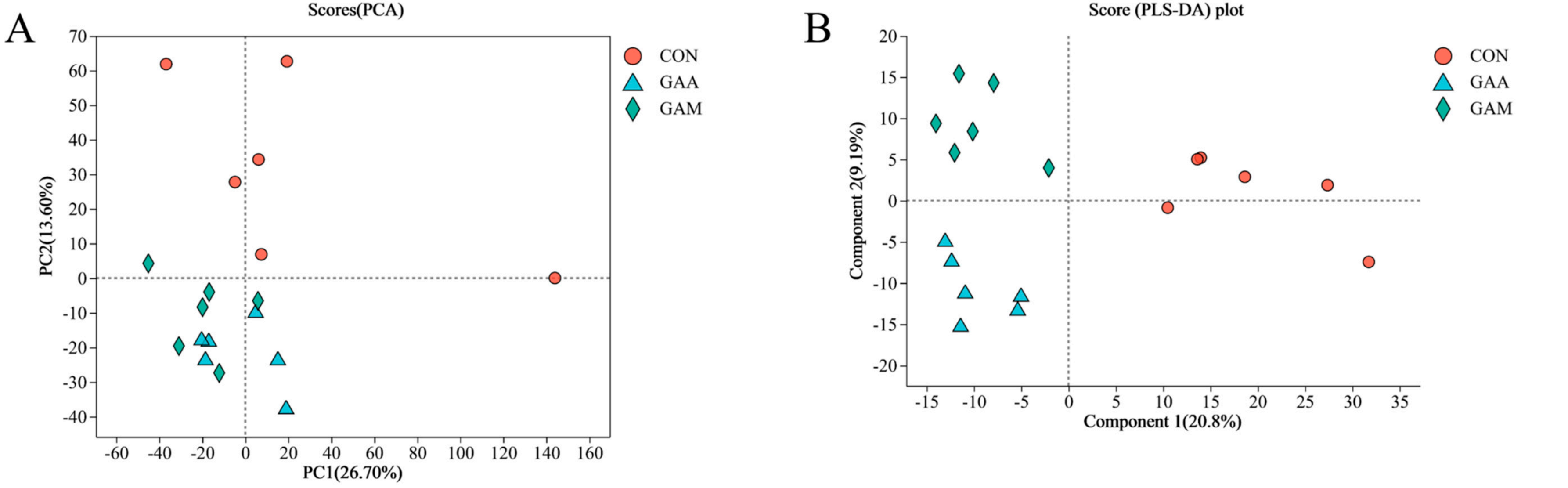

Figure S2. Sample relationship analysis diagram of liver metabolome. (**A**) PCA diagram; (**B**) PLS-DA diagram.
