# Supplementary material for: Guanidinoacetic Acid and Methionine Supplementation Improve the Growth Performance of Beef Cattle via Regulating the Antioxidant Levels and Protein and Lipid Metabolisms in Serum and Liver
Source: Antioxidants (Basel). 2025 May 8;14(5):559. doi: 10.3390/antiox14050559 (PMC12108366; doi:10.3390/antiox14050559)
Supplement: Supplementary file 1 [file antioxidants-14-00559-s001.zip › antioxidants-3513798-supplementary/Table S1.pdf]

Table S1 The composition and nutritional level of the diet (DM basis, %)

Items	content
**Ingredient composition** (% DM basis)	
Whole Corn Silage	29.1
Corn stalks	10.9
Corn	30.0
Jujube meal	8.6
Soybean meal	7.5
Palm meal	7.5
$NaHCO_3$	1.2
NaCl	1.0
Premix[1]	4.2
Total	100.00
**Chemical composition** (DM basis)[2]	
DM, %	95.48
CP, %	11.50
NDF, %	42.74
Ash, %	10.58
Ca, %	0.69
P, %	0.43
ME/(Mcal/kg)	2.49
NE_m/(Mcal/kg)	1.62
NE_g/(Mcal/kg)	1.04

[1]The premix provided the following per kg of diets: Cu 10 mg, Fe 65 mg, Mn 30 mg, Zn 25 mg, I 0.5 mg, Se 0.1 mg, Co 0.1 mg, V_A 4 000 IU, V_D 500 IU, and V_E 40 IU.

[2]DM: dry matter; CP: crude protein; NDF: neutral detergent fibres; Ca: calcium; P: phosphorus. ME, NE_m and NE_g were calculated with the equations proposed by the NASEM (2016).
