# Supplementary material for: Guanidinoacetic Acid and Methionine Supplementation Improve the Growth Performance of Beef Cattle via Regulating the Antioxidant Levels and Protein and Lipid Metabolisms in Serum and Liver
Source: Antioxidants (Basel). 2025 May 8;14(5):559. doi: 10.3390/antiox14050559 (PMC12108366; doi:10.3390/antiox14050559)
Supplement: Supplementary file 1 [file antioxidants-14-00559-s001.zip › antioxidants-3513798-supplementary/Table S2.pdf]

Table S2 Statistics of total ion number and metabolite number

Items	Content
Ion mode	Mix
All peaks	6190
Identified metabolites	1251
Metabolites in Library	1187
Metabolites in KEGG	686

Note: (1) Ion mode: the ion mode of the substance detected by the mass spectrometer, mainly: pos (positive ion mode) and neg (negative ion mode); (2) All peaks: the number of peaks extracted by the software; (3) Identified metabolites: the number of metabolites finally identified by the mass spectrometry data of the first and second level, searching the libraries (self-constructed libraries, Metlin (3) Identified metabolites: the number of metabolites finally identified by searching library (self-built library, Metlin, HMDB, etc.) through the primary and secondary mass spectrometry data; (4) Metabolites in library: the number of metabolites annotated to public databases such as HMDB and Lipidmaps; (5) Metabolites in kegg: the number of metabolites annotated to the KEGG database.
